# A 3D Heterotypic Breast Cancer Model Demonstrates a Role for Mesenchymal Stem Cells in Driving a Proliferative and Invasive Phenotype

**DOI:** 10.3390/cancers12082290

**Published:** 2020-08-14

**Authors:** Amarnath Pal, Jennifer C. Ashworth, Pamela Collier, Catherine Probert, Sal Jones, Eduardo Pernaut Leza, Marian L. Meakin, Alison A. Ritchie, David Onion, Philip A Clarke, Cinzia Allegrucci, Anna M. Grabowska

**Affiliations:** 1 Ex Vivo Cancer Pharmacology Centre, Division of Cancer and Stem Cells, Biodiscovery Institute, School of Medicine, University of Nottingham, Nottingham NG7 2UH, UK; amar.250357@gmail.com (A.P.); jennifer.ashworth@nottingham.ac.uk (J.C.A.); pamela.collier@nottingham.ac.uk (P.C.); catherine.probert@nottingham.ac.uk (C.P.); Sal.Jones@nottingham.ac.uk (S.J.); eduardo.pernaut-leza@nottingham.ac.uk (E.P.L.); marian.meakin@nottingham.ac.uk (M.L.M.); alison.ritchie@nottingham.ac.uk (A.A.R.); phil.clarke@nottingham.ac.uk (P.A.C.); 2 Flow Cytometry Facility, School of Life Sciences, University of Nottingham, Nottingham NG7 2UH, UK; david.onion@nottingham.ac.uk; 3 SVMS, Biodiscovery Institute, University of Nottingham, Nottingham NG7 2RD, UK; cinzia.allegrucci@nottingham.ac.uk

**Keywords:** spheroid co-culture model, tumour stroma, mesenchymal stem cells (MSCs), tumour microenvironment (TME), epithelial-mesenchymal transition (EMT), ski-related novel protein N (SnON), Wnt signalling, TGF-β signalling

## Abstract

Previous indirect 2D co-culture studies have demonstrated that mesenchymal stem cells (MSCs) promote breast cancer (BC) progression through secretion of paracrine factors including growth factors, cytokines and chemokines. In order to investigate this aspect of the tumour microenvironment in a more relevant 3D co-culture model, spheroids incorporating breast cancer cells (BCCs), both cell lines and primary BCCs expanded as patient-derived xenografts, and MSCs were established. MSCs in co-cultures were shown to enhance proliferation of estrogen receptor (ER)/progesterone receptor (PR)-positive BCCs. In addition, co-culture resulted in downregulation of E-cadherin in parallel with upregulation of the epithelial-mesenchymal transition (EMT)-relation transcription factor, SNAIL. Cytoplasmic relocalization of ski-related novel protein N (SnON), a negative regulator of transforming growth factor-beta (TGF-β) signalling, and of β-catenin, involved in a number of pathways including Wnt signalling, was also observed in BCCs in co-cultures in contrast to monocultures. In addition, the β-catenin inhibitor, 3-[[(4-methylphenyl)sulfonyl]amino]-benzoic acid methyl ester (MSAB), mediated reduced growth and invasion in the co-cultures. This study highlights the potential role for SnON as a biomarker for BC invasiveness, and the importance of interactions between TGF-β and Wnt signalling, involving SnON. Such pathways may contribute towards identifying possible targets for therapeutic intervention in BC patients.

## 1. Introduction

There is growing recognition of the importance of the tumour microenvironment (TME), especially cells within the stroma, in determining biological characteristics of cancer cells such as proliferation, invasion and drug resistance [1,2,3,4,5,6]. Drugs specifically targeting the stroma are being investigated [7], but appropriate models are required in order to identify relevant targets. 

During cancer development, there is active recruitment of mesenchymal stem cells (MSCs) from bone marrow to the TME where MSCs are educated by cancer cells to form cancer-associated fibroblast (CAF)-like cells [8]. The presence of several growth factors and chemokines including hepatocyte growth factor (HGF), monocyte chemotactic protein-1 (MCP-1), interleukin-6 (IL-6), transforming growth factor-beta (TGF-β) and CCL-5 in MSC-conditioned medium in 2D suggests that, once within the TME, MSCs secrete growth factors that promote tumour growth, epithelial-mesenchymal transition (EMT) and invasion through direct paracrine actions and remodelling of extracellular matrix [8,9,10,11]; thus, activated signalling axes identified in this way may provide therapeutic targets. 

However, studying the impact of MSC-driven paracrine signalling on BC progression in 2D may not be an ideal approach. In 3D, extracellular matrix (ECM) supports cells through focal adhesion and participates in cell signalling by promoting interaction between growth factors and cell surface receptors [12,13]. In addition, in 2D cell culture systems, lower numbers of gap junctions prevent exchange of ions and secondary metabolites and block removal of waste materials [14]. Alternatively, in vivo xenograft models have been used [15] but cross-species interaction between human tumour and murine stromal cells [16] may mask the real signalling axes activated in cancer cells by human MSCs.

In contrast, 3D spheroids potentially provide a useful system for modelling heterotypic interactions, overcoming some of these problems, and are increasingly being used for drug screening and drug penetration studies [17,18,19,20]. In spheroids, cells grow in aggregates that result in cell–cell interactions and, under some conditions, the nutrition and oxygen gradients observed in real tissue [21]. In addition, potential for incorporation of ECM facilitates cell–matrix interaction [22]. 

A high stroma–tumour ratio has been shown to be a poor prognostic indicator for breast cancer patient overall survival and distant metastasis-free survival [23]. Uncontrolled cell proliferation is an indication of the onset of neoplasia and a risk factor in patients [24,25], while epithelial-mesenchymal transformation (EMT) facilitates invasion [26]. Therefore, understanding the molecular mechanisms underlying breast cancer progression and the role of the stroma in driving it is important.

Hence, we chose to investigate the potential of using spheroid co-culture to determine the influence of MSCs on breast cancer cell (BCC) proliferation and acquisition of an invasive phenotype. We have studied paracrine signalling, which together with juxtracrine interactions plays an important role in the tumour microenvironment and induction of EMT [27,28]. CAFs secrete various growth factors such as TGF-β, HGF, FGF and chemokine CXCL12/SDF-1 (C-X-C motif chemokine ligand 12 or Stromal cell-derived factor 1), which together promote tumour growth, EMT and invasion in cancer cells [4]. Here, we have focused on components of two pathways, known to be involved in crosstalk between paracrine signalling axes in order to illustrate the potential applications of this model system and to explore the contribution of these factors in BC. 

β-catenin is a well-known component of the Wnt pathway [29] but also serves as a downstream molecule for EGF/AKT and CCL5 signalling axes [30,31] and, in its non-phosphorylated form, is transcriptionally active enhancing expression of Jun, c-Myc and Cyclin-D [32]. Increased cytoplasmic and nuclear levels of β-catenin are found in the S and G2/M phases of proliferating cells [33], and nuclear localisation of β-catenin leads to activation of cyclin-D1, important in regulation of the cell cycle in colorectal cancer [34].

TGF-β plays an important role in EMT and metastasis in cancer cells [35]. It regulates several signalling axes including Extracellular signal-regulated kinase (ERK), p38, mitogen-activated protein kinase (MAPK), c-Jun N-terminal kinase (JNK), Phosphatidylinositol-3-kinase (PI3)-protein kinase B (PKB, also known as AKT), acting primarily as a tumour suppressor in premalignant epithelial cells but switching to an EMT promoter as cancer evolves [36]. Ski-related novel protein N (SnON) antagonises the TGF-β signalling pathway via binding to the Smad protein and interrupts its interaction with co-activators [37]. 

Thus, given the known crosstalk between TGF-β signalling and other pathways including Wnt [11], the association of β-catenin with increased proliferation and poor survival in BC patients [38] and the observation that shRNA-mediated knockdown of SnON has been shown to increase migration and invasion in breast and lung cancer cells [39]. The involvement of β-catenin and SnoN downstream of MSC-mediated paracrine signalling was investigated in a spheroid co-culture model of breast cancer.

## 2. Results

### 2.1. MSCs Promote Proliferation in MCF-7 Breast Cancer Cells in Spheroid Co-Cultures

**Figure 1:**
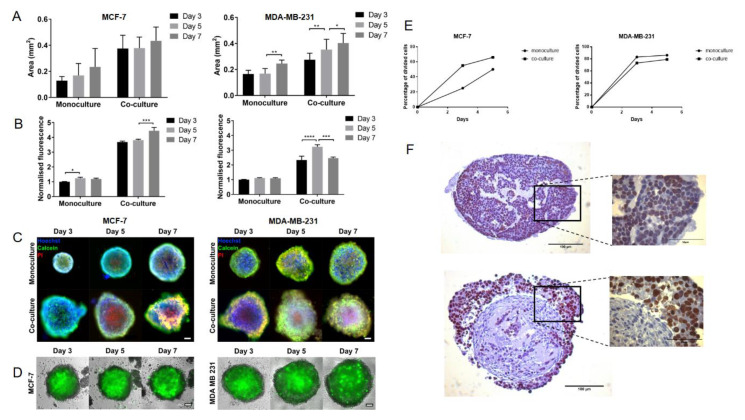
Mesenchymal stem cells (MSCs) promote proliferation of breast cancer cells (BCCs) in spheroid co-culture. BCC cancer cells (MCF-7 or MDA-MB-231) were cultured as spheroids alone (monoculture) or MSCs at a 1:2 (cancer cells: MSCs) ratio (co-culture). (**A**–**C**) The spheroids were analysed at d3, d5 and d7 after initiation to determine projected area (**A**) based on image analysis of bright field micrographs captured at each time point from three independent replicates; relative cell number (**B**) was assessed at each time point using the AlamarBlue assay; live/dead cell staining (**C**) was carried out using Calcein acetoxymethyl (AM) and Propidium iodide (PI) respectively, together with Hoechst staining to localise individual cells. Images shown represent a single z-slice captured using 10× magnification. (**D**) Spheroids were additionally formed using enhanced green fluorescent protein (eGFP)-labelled MSCs in order to investigate MSC localisation and retention in the co-cultures. Fluorescent images shown represent a single z-slice captured using fluorescent microscopy. (**E**) To investigate proliferation in the cancer cells directly, spheroids were established using BCCs that had been labelled with Cell-trace violet, then disaggregated and analysed by flow cytometry. The proportion of cancer cells which had divided between initiation of spheroids and d3 or d5 of culture was calculated (see Supplementary Figure S1 for details) using Weasel flow cytometry-analysis software. (**F**) Following fixation and paraffin-embedding, MCF-7 spheroids harvested at d5 were immuno-stained using anti-Ki-67 antibody. Brown colour indicates the presence of the Ki-67-positive nuclei. Images were captured at 20× and 60× magnifications using a Leica DFC480 digital microscope. Statistical significance between consecutive time points is shown for each condition, based on two-way repeated measures ANOVA: *, **, *** and **** represent *p*-values of <0.05, <0.01, <0.001 and <0.0001 respectively. Scale bars represent 100 µm (**C**,**D**,**F**) or 50 µm (F inset).

Initially, a number of alternative approaches were taken in order to assess co-culture models and to identify models suitable for further use. The growth of spheroid co-cultures of luminal (MCF-7) and triple negative (MDA-MB-231) cells with MSCs as well as monoculture controls (MCF-7 or MDA-MB-231 only) was investigated using spheroid projected area measurements (Figure 1A) or Alamar Blue assays (Figure 1B). Given the higher numbers of cells in the co-cultures, there were higher signals in in the co-culture models compared with monocultures at all timepoints assessed (d3, d5 and d7). Additionally, although not apparent from the area measurements, there was a small reduction in viability observed in some of the models at d7 compared to d5. Live/dead staining using Calcein acetoxymethyl (AM) and propidium iodide (PI) confirmed the slight reduction in cell viability at later timepoints, particularly in the core of the spheroids (Figure 1C). To determine the location of MSCs in the co-cultures and their retention over time, enhanced green fluorescent protein (eGFP)-labelled MSCs were used to establish spheroids (Figure 1D). The eGFP signal was strongest in the core of the spheroids, suggesting that the small population of dying cells in the core of the spheroids based on Calcein AM/PI staining consisted of MSCs. 

Since the area measurements, AlamarBlue and live-dead staining approaches do not distinguish between signals derived from cancer cells and MSCs, a more direct approach to measure proliferation in the cancer cells was taken. CellTrace Violet diffuses into cells, where it is cleaved by intracellular esterases to yield a highly fluorescent compound shared between daughter cells on cell division, resulting in a distinct peak separate from that in the parental cells that can be detected by flow cytometry analysis [40]. Details of the analysis are shown in Figure S1, and the results are summarised in Figure 1E. Co-culture of MDA-MB-231 with MSCs had little effect on their rate of division (73% vs. 85% at d3 and 79% vs. 86% at d5). However, for the MCF7 cells, there was a notable increase in the number of dividing cells in the co-culture compared with the monoculture, especially at d3 (73% vs. 15%). 

To confirm and localise the proliferating BCCs in the MCF-7 co-cultures, paraffin-embedded sections of microarrayed MCF-7 mono and co-culture spheroids were probed for Ki-67. In the monoculture spheroids, a few Ki67-positive cells were seen and, in general, the Ki67-positive nuclei were only weakly stained; interestingly, low levels of Ki67 have previously been observed in breast cells at the G1/S transition in slowly cycling cells, suggesting that low intensity as well as low Ki67 counts are associated with cells which are not proliferating [41]. In the MCF-7 co-cultures, a large number of brightly stained, Ki67-positve nuclei were observed (Figure 1F). Many of these were in the zone outside the core where a low eGFP signal was observed in the BCC:MSC-eGFP co-culture spheroids. Within and particularly at the edges of the core, mesenchymal-like cells, likely to be MSCs, were observed which were rarely Ki67-positive; however, some epithelial cancer cells were also observed within the core, many of which were Ki67-positive. Thus, this confirmed the CellTrace Violet data, which suggests that it is mainly the BCCs within the MCF-7 co-culture spheroids which are proliferating.

### 2.2. MSCs Promote Invasion in Noninvasive BC Cell Line MCF-7

**Figure 2:**
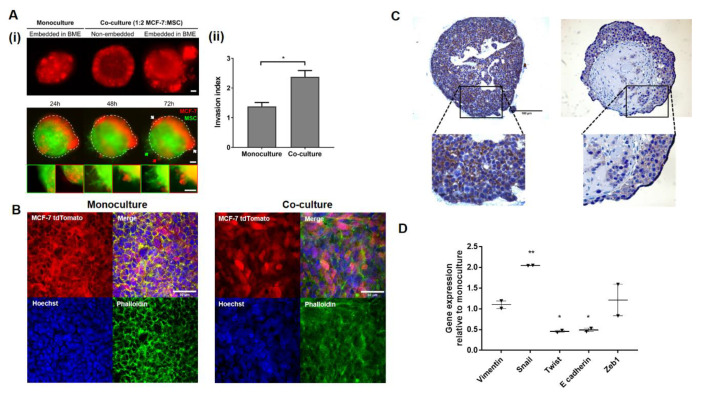
Mesenchymal stem cell (MSC)-induced invasion, cytoskeletal organisation and epithelial-mesenchymal transition (EMT) in MCF-7 spheroid co-cultures. MCF-7s spheroid monocultures, or co-cultures with MSCs were embedded in high-gelling basement membrane extract (BME, 3 mg/mL) to investigate invasion and associated phenotypic markers. Non-embedded spheroids were examined as controls. (**A**) Spheroids formed using tdTomato and eGFP-labelled MCF-7s and MSCs respectively were imaged at 24, 48 and 72 h. (i) Cancer cells (red) within co-cultures embedded in BME or controls at 72 h are shown in the upper panel. Images in the lower panel indicate that some MSCs are also invading. Regions of localised spheroid invasion are shown as insets in the lower panel. Scale bar 100 µm. (ii) Based on red fluorescence measurements, invasion of MCF-7 cells was quantified at 72 h as invasion index (perimeter/circumference). Statistical significance was assessed using a Student’s *t*-test, * indicates *p* < 0.05. (**B**) Staining with Phalloidin to investigate the impact of MSCs on F-actin reorganisation (green) in MCF-7 at the outer edges of the spheroids. Maximum intensity projections of 5 z-slices obtained from confocal imaging using a Leica TCS SPE system are shown. Scale bar 50 µm. (**C**) immunohistochemistry (IHC) for E-cadherin was performed on 5 µm sections of paraffin-embedded, arrays of spheroid mono and co-cultures. (**D**) Relative expression (2-∆∆Ct) of EMT markers (mean±SEM in two independent replicates) was assessed by qRT-PCR in FACS-sorted MCF-7 tdTomato cells from spheroid co-culture. Significant changes in expression relative to the control monoculture are indicated (* *p* < 0.05 and ** *p* < 0.01).

Using a concentration of basement membrane extract (BME, 3 mg/mL) that supports the invasion of triple negative breast cancer (TNBC) MDA-MB-231 monoculture spheroids, the impact of mesenchymal cells on the invasive phenotype of MCF-7tdTomato in the presence or absence of MSCs was assessed. In the case of spheroid co-cultures, mobility of the cancer cells beyond the original boundaries of the spheroid was observed (Figure 2Ai) and was significantly higher (*p* < 0.05) in the co-cultures compared with monocultures (Figure 2Aii). In contrast, no such migrated MCF-7tdTomato cells were observed either in embedded monoculture spheroids or in non-embedded spheroid co-cultures (Figure 2Ai). Some invasion of eGFP-labelled MSCs into the surrounding matrix was also observed (Figure 2Ai, lower panel).

In the MCF-7 spheroid co-cultures, cytoskeletal reorganisation was also observed. Phalloidin staining of spheroid monoculture showed a honeycomb structure associated with well-differentiated epithelial cells with F-actin at the plasma membrane surrounding the cells (Figure 2B). In contrast, rearrangement of F-actin occurred in MCF-7 with loss of the honeycomb pattern in spheroid co-cultures as previously observed in mammary epithelial cells treated with TGF-β [42]. Since such F-actin reorganisation was also associated with EMT [42], expression of E-cadherin was assessed by immunohistochemical staining of paraffin-embedded spheroids and further confirmed the loss of epithelial characteristics in MCF-7 cells in co-culture (Figure 2C). Furthermore, the gene expressions of EMT markers vimentin, SNAIL, TWIST, E-cadherin and Zeb1 were investigated in MCF-7-tdTomato cells FACS-sorted from co-culture spheroids and compared with that in monoculture spheroids (Figure 2D). Surprisingly, the expressions of vimentin and Zeb1 were unchanged and there was a significant reduction in TWIST expression. However, consistent with the protein expression, E-cadherin gene expression was significantly downregulated in the co-culture (*p* < 0.05, paired *t*-test). This was paralleled by significant upregulation of SNAIL expression (*p* < 0.01, paired *t*-test). 

### 2.3. MSCs Induce Proliferation and Promote Invasion in Estrogen Receptor (ER)/Progesterone Receptor (PR)-Negative PDXs in Spheroid Co-Culture

To further assess the effects of MSCs on BCC proliferation and invasion in 3D models using patient-relevant cells, BCC cells derived from breast cancer patient-derived xenograft (PDX) samples were used in the spheroid co-culture models. BR15 derived from an ER/PR-positive patient tumour sample and BR8 derived from an ER/PR-negative patient tumour sample associated with lymph node and brain metastasis were used. 

**Figure 3:**
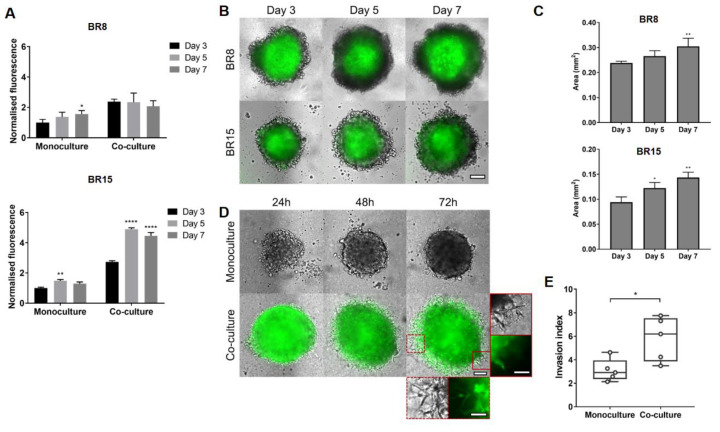
Mesenchymal stem cells (MSCs) promote proliferation in breast cancer cells isolated from patient-derived xenografts (PDXs) and induce invasion in a non-invasive estrogen receptor (ER)/progesterone receptor (PR)-negative PDX in spheroid co-culture. Spheroid monocultures and co-cultures of PDXs BR8 (ER/PR-negative) and BR15 (ER/PR-positive) were established. (**A**) AlamarBlue was used to assess viability on days 3, 5 and 7. Statistical significance was determined using two-way repeated measures ANOVA with Sidak post-hoc test, shown relative to day 3. (**B**) Fluorescence imaging was used to assess location and survival of MSC-eGFP, scale bar 100 µm. (**C**) Total spheroid projected area for the co-cultures was measured from bright-field images at each time point. Statistical significance was determined using one-way ANOVA with Tukey post-hoc test, shown relative to day 3. (**D**) For BR15, the effect of co-culture with MSCs on invasion into BME was assessed using Brightfield and Fluorescent microscopy at 10x magnification. Scale bar 100 µm, 50 µm inset. (**E**) Quantification of invasion index (perimeter/circumference) for BR15 invasion at 72 h for monoculture and co-culture. Statistical significance was determined using the Student’s *t*-test. *, ** and **** indicate *p*-values of <0.05, <0.01 and <0.001 respectively.

As observed in the cell-line spheroid models, there was growth over time but a slight reduction in overall cell viability by day 7 (Figure 3A) and a stronger effect in the ER positive BR15 model compared to triple negative BR8. In addition, MSCs tended to cluster in the centre of the spheroids (Figure 3B) and there was good retention of the MSCs over time (Figure 3C). We next investigated the effect of co-culture with MSCs on invasion of non-invasive BR15 spheroids, following embedding in BME. Although the edges of the BR15 spheroid monocultures remained regular, finger-like projections were observed in the spheroid co-culture starting from 48 hours (Figure 3D), which is consistent with the results observed in the MCF-7 in the spheroid co-culture (Figure 2A) with statistically significantly higher invasion in the co-cultures compared with the monocultures (Figure 3E).

### 2.4. MSCs Promote Nuclear Clearance of SnON and Promote β-Catenin Activation in MCF-7 in Spheroid Co-Culture 

**Figure 4:**
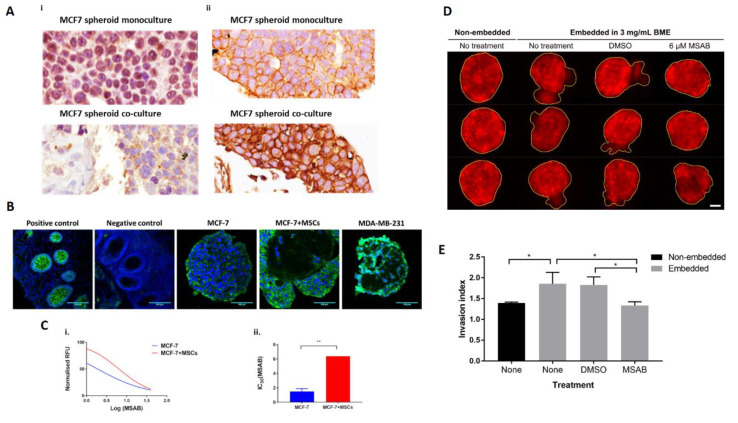
Mesenchymal stem cells (MSCs) induce a cytoplasmic shift of ski-related novel protein N (SnON) and promote BCC progression through β-catenin activation. MCF-7 monoculture and co-culture spheroids were established and examined for expression of SnON and β-catenin, and the effect of β-catenin inhibition on spheroid growth and invasion was assessed. MDA-MB-231 monoculture spheroids were used as controls. (**A**) IHC staining for SnON (i) and β-catenin (ii). Brown 3,3′-Diaminobenzidine (DAB) indicates the location of the target proteins; images were captured at 60× objective using a slide scanner, Ventana 2000. (**B**) Confocal images of fluorescent staining of β-catenin captured using a 20× objective. β-catenin appears green while 4′,6-diamidino-2-phenylindole (DAPI stained cell nuclei blue. Colon tissue was used as a positive control. (**C**) AlamarBlue assay was used to assess the effect of β-catenin inhibitor on MCF-7 spheroids (i) and the mean ±SEM IC50 values calculated (ii). Statistical significance between effects on monoculture and co-culture is indicated (** *p* < 0.01; *t*-test, *n* = 2). (**D**) Invasion of spheroid co-cultures embedded into 3 mg/mL BME was imaged at 72 h at 10× magnification. Spheroids treated with 6 μM 3-[[(4-methylphenyl)sulfonyl]amino]-benzoic acid methyl ester (MSAB) were compared with non-embedded, untreated or control-treated spheroids. The invasive front in each condition is highlighted using a yellow border drawn in ImageJ. (**E**) The invasion index (perimeter/circumference) under each condition was quantified. Significant differences were calculated using one-way ANOVA with the Tukey post-hoc test (* indicates *p* < 0.05).

To study the mechanism of induced proliferation and invasion by MSCs in BCCs, we focus our attention on the crosstalk between TGFβ and Wnt signalling as known effectors of the tumour microenvironment on BCC behaviour and inducers of EMT [43]. We first studied the expression of SnON, a known negative regulator of TGFβ signalling [44]. SnON is mainly a nuclear protein but becomes more cytoplasmic in ER/PR-negative grade III ductal breast tumours with high expression of Ki67 and HER2 amplification [45], representing a more aggressive subtype of BC. Similarly, it has been reported that cytoplasmic and nuclear levels of β-catenin increase in the S and G2/M phases of proliferating cells [33]. Since co-culture with MSCs increased the expression of Ki-67 and invasion in MCF-7 cells, immunohistochemistry (IHC) for SnON and β-catenin expression was performed and revealed nuclear localisation of SnON in MCF-7 in the spheroid monoculture, while cytoplasmic expression of SnON in MCF-7 in co-culture spheroids imitates the SnON localisation in the cytoplasm of MDA-MB-231 (Figure 4Ai). On the other hand, cytoplasmic localisation of β-catenin in MCF-7 in spheroid co-culture contrasted with its presence at the plasma membrane of cells demonstrated in monocultures (Figure 4Aii). Controls for the SnON and β-catenin staining are shown in Figure S2. Moreover, from visual comparison of fluorescence staining intensity for β-catenin in spheroid mono- and co-culture of MCF-7, it appears that the presence of MSCs stabilizes β-catenin in co-culture (Figure 4B). 

Since changes in the distribution of β-catenin observed by IHC and fluorescent staining suggested a possible role for β-catenin in BC progression, the effect of β-catenin inhibition on MCF-7 cells in spheroid co-culture was investigated using MSAB [46]. Spheroid mono and co-culture were treated with a range of concentrations of MSAB (1 μM to 40 μM) for 48 h and viability assessed by Alamar blue assay, ensuring that the concentration of dimethyl sulphoxide (DMSO) vehicle was at least 10% lower than the cytotoxic concentration (Figure S3). A dose-dependent decrease in cell proliferation was observed (Figure 4Ci) and inhibition of proliferation of the co-culture spheroids required a significantly higher MSAB concentration (*p* < 0.01) compared to the monoculture (Figure 4Cii). Application of MSAB at 6 µM (approx. IC50 based on viability assay) suppressed invasion of MCF-7 spheroid co-cultures embedded in matrix resulting in a rounded morphology similar to that of non-embedded co-cultures and controls (Figure 4D) and a significant reduction in invasion compared with the DMSO control (Figure 4E). 

## 3. Discussion

Using a spheroid model, we demonstrate the influence of bone-marrow-derived MSCs on BCC proliferation, EMT and invasion in direct co-culture. Importantly, these effects were observed in models established using primary BCCs expanded as PDXs as well as those using standard cell lines. We also highlight a potential mechanism underlying these observations involving SnON and β-catenin.

Although spheroid area, metabolic assays such as AlamarBlue, and live-dead staining can provide some indication of cell survival and proliferation within spheroids [47], interpretation of such data in direct co-culture models is more difficult. Instead, we used the CellTrace Violet assay, which enables labelling and tracking of the individual population of cells under investigation. This enabled demonstration of a specific effect of the MSCs on breast cancer cell proliferation, which was confirmed by immunohistochemistry staining of paraffin-embedded spheroids for Ki67. 

Additionally, we demonstrated enhanced invasive capacity in breast cancer cells directly co-cultured with MSCs in 3D. Again, analysis of the BCCs isolated by FACS from these direct co-cultures, together with immunohistochemistry staining, suggest that the normally noninvasive ER/PR-negative BCCs have reduced E-cadherin expression when co-cultured with MSCs. This is consistent with data from co-culture studies in which enhanced proliferation and induction of EMT was observed when colorectal cancer spheroids were subjected to indirect co-culture with fibroblasts [48] and with observations of crosstalk between multiple pathways capable of driving EMT [49]. 

In the current study, downregulation of E-cadherin was paralleled by upregulation of SNAIL expression. SNAIL is known to be a repressor of E-cadherin expression [49]. In liver fibrosis, EMT is induced via upregulation of SNAIL and is dependent on TGF-beta signalling from stromal cells [50]. In breast cancer, upregulation of SNAIL is associated with poor prognosis and tumour recurrence, and overexpression of SNAIL is sufficient to induce EMT in primary tumour cells in vitro and to increase recurrence in vivo [51]. Although TWIST and Zeb1 are also considered to be master regulators of EMT [52], we did not observe upregulated expression of these genes in our model. In some studies TWIST is described as being a regulator of SNAIL expression [49]; however, in other studies, no effect of TWIST overexpression in primary mammary epithelial cells on SNAIL was observed, suggesting that, at least in some contexts, these transcriptional regulators of EMT can function independently [53]. Additionally, as we did not observe marked changes in vimentin expression, the BCCs may be undergoing partial EMT in which epithelial and mesenchymal markers are expressed concurrently [54]. Interestingly, consistent with our observations of both cancer and mesenchymal cells at the invading front, partial EMT can support collective migration of cells including those with epithelial and mesenchymal phenotypes [55]. 

Given the role of TGF-β in cancer cell proliferation and EMT induction and the role of SnON as a negative regulator of TGF-β signalling [45], we hypothesised that it may be involved in the MSC-driven effects on proliferation and EMT that we had observed. Our observation of an association between a more invasive phenotype and the shift to cytoplasmic expression of SnON in MCF-7 when co-cultured with MSCs is interesting in light of increased cytoplasmic SnON associated with poor prognostic features in ductal breast cancer including higher Ki67 expression and loss of ER/PR [45], the latter usually associated with an invasive phenotype. Although the precise mechanism linking SnON with all of these features is not clear, it is known to interact with SMADs to prevent TGF-β signalling [44], and previous studies demonstrated TGF-β-driven downregulation of E-cadherin and F-actin reorganisation in a SnON-knockdown lung cancer cell line [39]. Thus, SnON may normally repress TGF-β signalling but relocalises to the cytoplasm in more invasive breast cancer, and our data suggest that this may be driven by other paracrine signals, including crosstalk between TGF-β signalling and other pathways.

Our data showing cytoplasmic localisation of β-catenin and MSAB inhibition of growth in spheroid co-cultures suggest the presence of active β-catenin in MCF-7s in the presence of MSCs. This may occur through β-catenin’s known effect on cell proliferation regulatory genes including C-MYC, Cyclin D and ID2 (inhibitor of DNA binding 2) [56]. Interestingly, β-catenin also interacts with SNAIL and drives EMT, characterized by F-actin polarization and activated by the ERK pathway [57]. Further, it has been shown to be directly involved in TGF-β-induced EMT via interaction between phosphorylated β-catenin and SMADs [58], again emphasizing the complex interplay between the paracrine signalling pathways driving stromal influences in cancer. In the future, this direct co-culture system will allow further investigation of the role of the tumour microenvironment driven by paracrine or juxtracrine signalling.

## 4. Materials and Methods 

### 4.1. Cell Culture Conditions

Wildtype BCCs, MCF-7 and MDA-MB-231 (obtained from the National Cancer Institute, USA, as part of the NCI-60 panel) or tdTomato-transduced MCF-7 (MCF-7tdTomato) were maintained in phenol red-free Roswell Park Memorial Institute (RPMI)-1640 medium supplemented with 10% foetal bovine serum (FBS) and 2 mM L-Glutamine (Sigma Aldrich, St. Louis, MO, USA) Wildtype or green fluorescent protein (GFP) gene-transduced bone marrow-derived mesenchymal stem cells (MSC or MSC-eGFP respectively) were maintained in mesenchymal stem cell medium (MSCM) supplemented with FBS and mesenchymal stem cell growth supplement (MSCGS) (ScienCell, Carlsbad, CA, USA). For both BCC lines, passages below 40 were used, while for MSCs, passage numbers 8 to 10 were used. Mycoplasma tests were performed regularly throughout the study to avoid using infected cells.

### 4.2. Patient-Derived Xenografts

Cells were isolated from breast cancer PDXs for use in spheroid models, using protocols previously established in our laboratory [59]. BR15 had been expanded from an ER/PR-positive breast tumour and no tumour was associated with the lymph nodes resected in parallel. BR8 was expanded from an ER/PR-negative tumour; a number of associated lymph nodes contained tumours, and a brain metastasis was present. Both were obtained as fresh surgical material from tumour resections at Nottingham University Hospitals NHS Trust, collected with informed patient consent and National Research Ethics Service (NRES) approval (NRES REC 10/H0405/6). Samples were used in accordance with NRES approval (NRES REC 08/H0403/37). Female CD-1 NuNu mice (8–10 weeks), purchased from Charles River UK and allowed to acclimatise for a week prior to use, and female Rag2^−/−^ γc^−/−^ (RAG2G, 8–10 weeks) bred in-house under PPL P375A76F, both immunodeficient strains, were used in this project. Mice were maintained in Individually Ventilated Cages (IVCs) (Tecniplast UK) within a barriered unit illuminated by fluorescent lights set to give a 12 hour light-dark cycle (on 07.00, off 19.00), as recommended in the United Kingdom Home Office Animals (Scientific Procedures) Act 1986. The room was air-conditioned by a system designed to maintain an air temperature range of 21 ± 2 °C and a humidity of 55% + 10%. Mice were housed in social groups during the procedure and provided with irradiated bedding and autoclaved nesting materials and environmental enrichment (Datesand, Bredbury, UK). Sterile irradiated 5V5R rodent diet (IPS Ltd, UK) and irradiated water (SLS, UK) was offered ad libitum. Tissue was generated by implantation of tumour fragments using an implant trochar (VetTech, Congleton, UK) into the mammary fat pad of the mice (RAG2G or CD1 nude mice-strain used in each case was determined by suitability and availability) with MSCs and Matrigel^TM^, followed by serial passage when needed, by experienced in vivo technicians under project license PPL 3003444. Tumours were measured weekly using Vernier calipers, and the volumes were calculated using the formula V = ab2/6, where a is the length and b is the width. Mice were also weighed weekly and checked daily by an experienced technician. National Cancer Research Institute (NCRI) guidelines for the welfare and use of animals in cancer research, Laboratory Animal Science Association (LASA) good practice guidelines, and Federation for Laboratory Animal Science Associations (FELASA) working group on pain and distress guidelines were also followed, as were the Animal Research: Reporting of In Vivo Experiments (ARRIVE) guidelines on the reporting of in vivo experiments.

### 4.3. Establishing Spheroid Mono and Co-Culture

Spheroids were established using either cell lines or cells isolated from PDXs; 2K BCCs were seeded alone for the spheroid monoculture, while for spheroid co-culture, 2K BCCs were mixed with 4K MSC (wild-type or eGFP) and seeded in a 96-well Ultra-Low Attachment plate (Corning, Corning, NY, USA) in a 200 µL volume per well. Extracellular matrix (ECM)-rich Cultrex (Trevigen Inc., Gaithersburg, MD, USA) was included at a final concentration of 100 µg/mL for all models as it was required to aid formation of tight round MDA-MB-231 spheroids. After centrifugation for at least 5 min at 300 Relative Centrifugal Field (RCF), the cultures were maintained in a cell culture incubator at 37 °C with 5% CO_2_ in a humidified atmosphere.

The cell culture medium used for PDX spheroids was high glucose DMEM media supplemented with 20% FBS, 2 mM L-glutamine, 1× antibiotic/antimycotic, 0.12% hydrocortisone and 0.09% insulin. 

In some experiments, spheroids were treated with 3-[[(4-methylphenyl)sulfonyl]amino]-benzoic acid methyl ester (MSAB, Sigma). Since DMSO is the solvent for MSAB and is toxic to cells, cytotoxic concentration of MSAB was determined by the Alamar blue assay while incubating MCF-7 spheroid monoculture with DMSO at the concentration gradient range from 0–5% (Figure S3).

### 4.4. Live/Dead Cell Staining in Spheroid Culture

Spheroids were incubated with 2 μM Calcein AM (eBioscienc, San Diego, CA, USA) and 5% (v/v) Propidium iodide (PI) (eBioscience, San Diego, CA, USA) [47] for 3 hours in the incubator in the presence of 5% CO_2_ at 37 °C and were washed twice with 300 μL PBS by carefully removing 100 µL of the media each time. Finally, fluorescent images of spheroids were captured using Nikon Eclipse TiE or Okolab fluorescence microscopes.

### 4.5. AlamarBlue Assay

Spheroids were incubated with 10% (v/v) AlamarBlue (Thermo Fisher Scientific, Waltham, MA, USA) for 90 min in the cell culture incubator, and the relative fluorescence (RFU) at different time points was measured with the assistance of a plate reader (Fluostar Omega, BMG Labtech, Ortenberg, Germany; FlexStation II, Molecular Devices, San Jose, CA, USA) at excitation and emission wavelengths of 544 nm and 590 nm, respectively. Data shown has been normalized to the values for monoculture day 3. For drug inhibition studies, the IC50 was calculated from the nonlinear drug response curve using GraphPad Prism.

### 4.6. CellTrace Violet Assay

CellTrace Violet assay was performed according to the manufacturer protocol (ThermoFisher Scientific, Waltham, MA, USA). Stained BCCs were seeded as above for spheroid mono and co-culture with MSCs. In order to demonstrate the BCC proliferation in different conditions, the spheroid mono and co-cultures from day 3 and day 5, respectively, were dissociated using Accumax (Sigma, St. Louis, MO, USA) (Figure S4) and the progressive decrease in signal intensity assessed by flow cytometry at excitation and emission wavelengths of 405 nm and 450 nm, respectively, and then by using Weasel software.

### 4.7. Spheroid Invasion Assay

Following formation of spheroids, the surrounding media was carefully removed from each well and replaced with 100 µL of 3 mg/mL Cultrex in complete medium per well. After incubating for 1 h at 37 °C when the gel had set, complete medium was overlaid on top and images captured as required at different time points.

### 4.8. Phalloidin Staining in Spheroid Mono and Co-Culture

Phalloidin staining was based on a previous study [60] but with minor modifications. Individual spheroids were stained with 1× phalloidin (Phalloidin-iFluor 488 Reagent-Cytopainter; Abcam, Cambridge) in an Eppendorf tube and embedded in polymerised high-gelling Cultrex (6 mg/mL) within a hydrophobic barrier on a glass slide for imaging. Stained spheroids were imaged using a Leica DMI4000B inverted microscope with a Leica TCS SPE laser scanning confocal setup.

### 4.9. RNA Extraction from FACS-Sorted MCF-7td Tomato in Spheroid Co-Culture and qRT-PCR 

Following digestion of eighty spheroids, cells were FACS sorted and total RNA from eighty spheroids from each condition was extracted using Trizol/chloroform procedure. Briefly, cell pellets of MCF-7tdTomato from each condition were treated with 300 μL Trizol; 250 μL chloroform was added with shaking for 5 min; and then, after a 10 min incubation at room temperature (RT), it centrifuged at 12,000× *g* for 15 min at 4 °C. The aqueous phase was mixed with an equal volume of ice-cold isopropanol in fresh centrifuge tubes and, then after 15 s of vigorous shaking, incubated for overnight at −20 °C. Samples were centrifuged at 12,000 × *g* for 20 min at 4 °C to pellet the RNA, which was washed twice in 75% ethanol and then resuspended in 8 μL Nuclease free water (NFW). The concentration of RNA in each sample was determined using Nanodrop (NanoDrop 2000, Thermo Fisher Scientific, Waltham, MA, USA).

cDNA prepared following the manufacturer’s protocol (GoSript™ Reverse Transcriptase) was analysed by real-time PCR using the Power SYBR Green PCR master mix (Applied Biosystems) using Hypoxanthine Phosphoribosyltransferase (HPRT) as an internal control in a StepOne PCR machine using the following cycles: 95 °C for 10 min, hot start for enzyme activation followed by 40 cycles at 95 °C for 15 s (denaturation) and 60 °C for 60 s (annealing/extending). Primer sequences (purchased from Eurofins, Luxembourg) used at a final concentration of 500 nM are shown in Table 1. 

### 4.10. Preparation and Immunostaining (IHC and IF) Analysis of Spheroid Microarray

Spheroids were washed three times with PBS and then fixed overnight at 4 °C in 4% paraformaldehyde (PFA). After two further PBS washes, they were embedded in 2% agarose, fixed in 1× Neutral buffer formalin (NBF) and paraffin embedded to prepare spheroid microarrays according to the published protocol [61].

Sections were cut, dewaxed and stained as before [61] except the primary antibody incubation was performed overnight at 4 °C. IHC and Immunofluorescence (IF) staining for β-catenin was performed following the protocol (Protocol ID: 283) provided by Cell Signalling Technology (CST). Details of antibodies and the secondary detection reagents used with each are given in Table 2.

### 4.11. Image Analysis

All image analysis was carried out using the ImageJ software distribution Fiji [62]. Raw data files were used for all quantitative analysis. The wand tool in Fiji was used to trace the spheroid perimeter and to determine the projected area. Imaging artefacts were removed manually where these were found to prevent accurate spheroid tracing. The measured area enclosed by the spheroid perimeter was used to calculate the spheroid radius, r, assuming a perfect circular cross-section, and area was calculated using the formula 2πr^2^. The same method was used for calculation of invasion index, defined as (spheroid perimeter)/2πr, which measures the departure of the shape from a perfect circle. Prior to tracing the spheroid perimeter for invasion analysis on bright field images, a “find edges” operation was carried out using the Fiji software. 

### 4.12. Statistical Analysis

Prism v.7.0d (GraphPad) and SPSS v.26 (IBM) were used for statistical analysis. All error bars shown represent mean and standard deviation unless otherwise indicated. To determine statistical significance, the Student’s *t*-test and one-way or two-way repeated measures ANOVA were used as appropriate. For multiple comparisons, the Tukey HSD and Sidak post hoc tests were used for one-way and two-way ANOVA, respectively. Statistical significance for all tests was declared at *p* < 0.05.

## 5. Conclusions

To restore mesenchymal cell-driven signalling, we have established a co-culture model incorporating BCCs together with MSCs, using either BC cell lines or BCCs isolated from patient-derived xenografts. Using these models, we have shown that there is enhanced proliferation and invasion of ER/PR-positive BCCs in the co-culture models compared with the monocultures. Care must be taken in interpreting effects on spheroid size, metabolic assays and even live/dead staining in co-culture models where the readouts are more complex due to the presence of multiple cell populations. Thus, we confirmed effects on proliferation using CellTrace Violet, which allows labelling and tracking of individual populations of cells, as well as through immunohistochemistry, which allows in situ localization of relevant markers.

The enhanced invasion observed when the co-culture spheroids were transferred into BME involves partial EMT, potentially mediated by upregulation of SNAIL and involving collective migration. As well as changes in EMT marker expression in the co-culture models, we observed relocalisation of TGF-β signalling repressor, SnON and β-catenin, suggesting that the phenotypic changes may occur via activation of TGF-β and Wnt signalling, a concept supported by inhibition of proliferation and invasion by a β-catenin inhibitor. Future studies may further elucidate the complex interactions between the multiple paracrine signalling pathways involved in mesenchymal cell-driven phenotypic changes in BCCs that result in disease progression. Direct co-culture models such as the one we have described here are important in allowing such studies to be carried out, reducing the use of animals to investigate such complex interactions. They also allow in vitro investigation using “close-to-patient” models such as the PDX cells utilized here, which will allow more robust and clinically relevant identification of novel drug targets and drug screening. 

## Figures and Tables

**Table 1:** Quantitative Real-time PCR primers for epithelial-mesenchymal transition (EMT) markers.

Gene	Forward Primer (5′-3′)	Reverse Primer (5′-3′)
HPRT	ATTATGCTGAGGATTTGGAAAGGG	GCCTCCCATCTCCTTCATCAC
Vimentin	AAAACACCCTGCAATCTTTCAGA	CACTTTGCGTTCAAGGTCAAGAC
Twist	CAAGCTGAGCAAGATTCAGACCC	AGACCGAGAAGGCGTAGCTGA
Snail	CCCCAATCGGAAGCCTAACT	GGTCGTAGGGCTGCTGCTGGAA
E-cadherin	GAACAGCACGTACACAGCCCT	GCAGAAGTGTCCCTGTTCCAG
Zeb1	GAAAGGAAGGGCAAGAAATCCT	TGCATCTGACTCGCATTCATC

**Table 2:** Antibodies used for IHC.

Antibodies	Dilution	Source
Monoclonal mouse (Mse) anti-Ki67 Ab (MIB-1)	1:40	DAKO
Monoclonal Mse anti-E-cadherin Ab (Clone NCH-38)	1:50	DAKO
Monoclonal mse anti-SnON (Clone OTI1A6)	1:150	LS Biosciences
β-catenin (D10A8) XP® rabbit monoclonal Ab	1:100	Cell Signalling Technology
Mse IgG1, Negative control	1:50	DAKO
Rabbit IgG1, Negative control	1:100	DAKO
Polyclonal rabbit anti-mouse IgG-Biotinylated	1:300	DAKO
SignalStain® Boost Detection Reagent (HRP, Rabbit)	neat	Cell Signalling Technology
Chicken anti-Rabbit IgG, Alexa Fluor 488	1:300	Thermo Fisher Scientific

## Supplementary Materials

The following are available online at https://www.mdpi.com/2072-6694/12/8/2290/s1, Figure S1: CellTrace Violet assay; Figure S2: Controls for SnON and β-catenin IHC staining; Figure S3: DMSO tolerance test, Figure S4: Accumax-mediated dissociation of spheroid.
