# Supplementary material for: A 3D Heterotypic Breast Cancer Model Demonstrates a Role for Mesenchymal Stem Cells in Driving a Proliferative and Invasive Phenotype
Source: Cancers (Basel). 2020 Aug 14;12(8):2290. doi: 10.3390/cancers12082290 (PMC7465555; doi:10.3390/cancers12082290)
Supplement: Supplementary file 1 [file cancers-12-02290-s001.pdf]

Supplementary Material: A 3D Heterotypic Breast Cancer Model Demonstrates a Role for Mesenchymal Stem Cells in Driving a Proliferative and Invasive Phenotype

Amarnath Pal, Jennifer C. Ashworth, Pamela Collier, Catherine Probert, Sal Jones, Eduardo Pernaut-Leza, Marian Meakin, Alison A. Ritchie, Philip A Clarke, Cinzia Allegrucci and Anna M. Grabowska

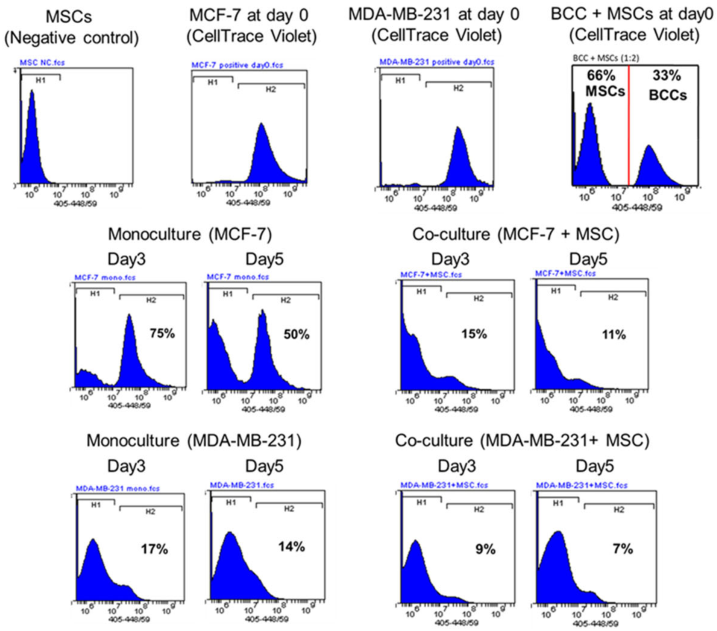

Cell-line	MCF-7			MDA-MB-231		
Timepoint	d0	d3	d5	d0	d3	d5
Mono-culture						
Percentage of +ve cells	100	75	50	100	17	14
Percentage of cells divided	0	25	50	0	83	86
Co-culture						
Percentage of +ve cells	33	15	11	33	9	7
Percentage of cells divided	0	55	66	0	73	79

Figure S1. CellTrace Violet assay. Breast cancer cells (MCF-7 and MDA-MB-231) stained with Cell-trace violet (excitation and emission wavelengths of 405 and 448/50 nm) were analysed by flow cytometry before seeding to form spheroid mono- and co-cultures. Spheroids were harvested on day 3 and day 5 respectively and digested using Accumax in order to prepare single cell suspensions for flow cytometry analysis. The H2 marker, based on the unstained control, was used to determine the proportion of positively stained cells in the spheroids compared to the initial cell populations.Since the co-cultures consisted of BCCs and MSCs at a ratio of 1:2, as expected, at initiation of the spheroid 33% of cells in the co-culture were positive for CellTrace violet. A reduction in the proportion of positive cells over time would indicate that the BCCs had divided. Thus, the proportion of positive cells was used to calculate the percentage of cells which have divided at a particular timepoint, which is given by the equation:

$$\frac{100 \times (\text{\%+ve cells at initiation} - \text{\%+ve cells at timepoint})}{\text{\%+ve cells at initiation}}$$

monocultures, 75% of the cells remained positive for CellTrace Violet at day 3 so (100 × (100 − 75))/100 = 25% of the original population had divided prior to this timepoint. In the MCF-7 co-culture at d3 the number of positively stained cells was 15% of the total population and 33% of the cells were positive at initiation, so (100 × (33 − 15)/33) = 55% of the MCF-7s had divided.

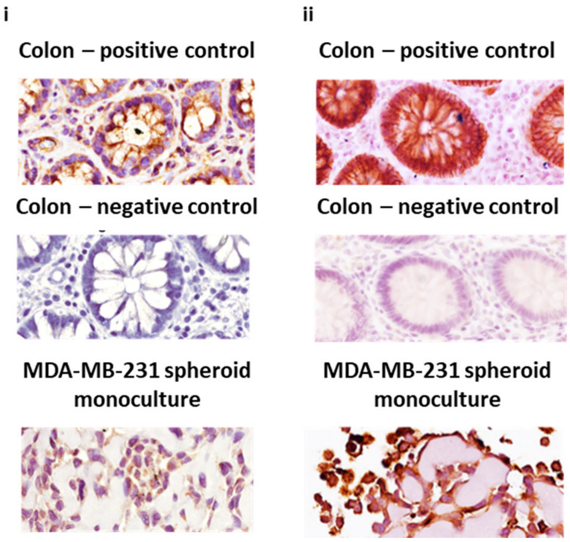

Figure S2. Controls for SnON and β-catenin IHC staining: Colon tissue was used to optimise (i) SnON and (ii) β-catenin IHC staining. In addition, results for staining of MDA-MB-231 spheroid monocultures are shown. Brown 3,3′-Diaminobenzidine (DAB) indicates the location of the target proteins; images were captured at 60× objective using a slide scanner, Ventana 2000.

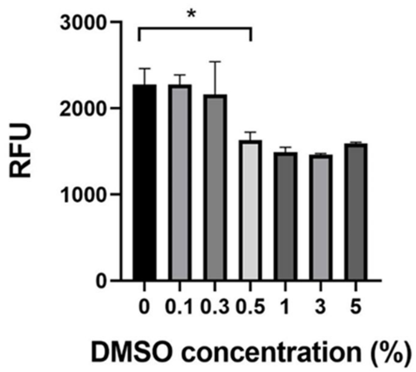

Figure S3. DMSO tolerance test: Spheroid monocultures of MCF-7 were treated with different concentrations of DMSO (0–5%) on day 3 and AlamarBlue assay was performed after 48 h. of treatment. Mean±SEM in the replicates demonstrated the significant difference (* p-value = 0.04) in cell viability between non-treated and DMSO treated monoculture.

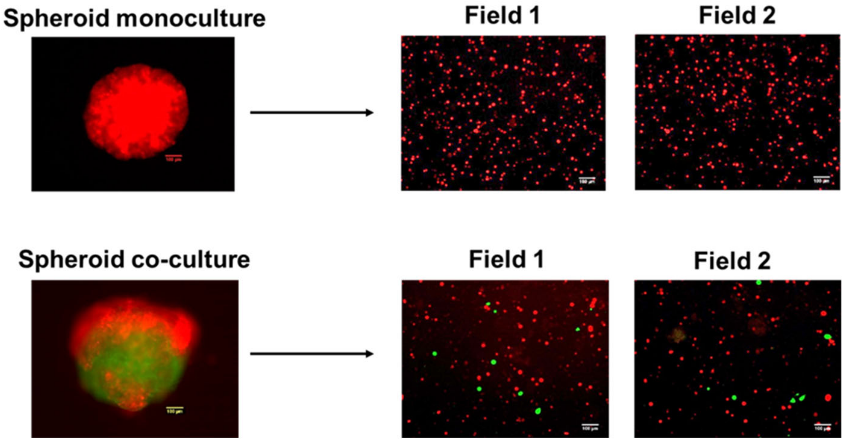

Figure S4. Accumax-mediated dissociation of spheroid: Representing Accumax-mediated dissociation of spheroids established with MCF-7tdTomato alone or in the presence of MSC-GFP for mono and co-culture respectively. Following post-digestion, fluorescence images of single cell suspensions from two different fields in each condition were captured at 10× magnification under compound fluorescence microscope. The method of spheroid dissociation was applied for flow cytometry-based assays including FAC-sorting of MCF-7tdTomato cells from mixed cell population and CellTrace Violet assay.

© 2020 by the authors. Licensee MDPI, Basel, Switzerland. This article is an open access article distributed under the terms and conditions of the Creative Commons Attribution (CC BY) license (http://creativecommons.org/licenses/by/4.0/).
